# Micronutrients and Renal Outcomes: A Prospective Cohort Study

**DOI:** 10.3390/nu14153063

**Published:** 2022-07-26

**Authors:** Chun-Yu Chen, Chun-Hui Chiu, I-Wen Wu, Heng-Jung Hsu, Yih-Ting Chen, Cheng-Kai Hsu, Heng-Chih Pan, Chin-Chan Lee, Chiao-Yin Sun

**Affiliations:** 1 Department of Nephrology, Chang Gung Memorial Hospital, Keelung 204, Taiwan; shone@cgmh.org.tw (C.-Y.C.); a22066@cgmh.org.tw (I.-W.W.); r5267@cgmh.org.tw (H.-J.H.); b9402031@cgmh.org.tw (Y.-T.C.); kylegb@cgmh.org.tw (C.-K.H.); balour@cgmh.org.tw (H.-C.P.); leefang@cgmh.org.tw (C.-C.L.); 2 College of Medicine, Chang Gung University, Taoyuan 333, Taiwan; 3 Community Medicine Research Center, Chang Gung Memorial Hospital, Keelung 204, Taiwan; 4 Department of Traditional Chinese Medicine, Chang Gung Memorial Hospital, Keelung 204, Taiwan; chchiu@mail.cgust.edu.tw; 5 Graduate Institute of Health Industry and Technology, Research Center for Chinese Herbal Medicine, Research Center for Food and Cosmetic Safety, College of Human Ecology, Chang Gung University of Science and Technology, Taoyuan 333, Taiwan

**Keywords:** micronutrient, chronic kidney disease, zinc, selenium, end stage renal disease

## Abstract

Background: Micronutrients are essential in maintaining normal human physiology. Data regarding the association between micronutrients and renal outcomes in chronic kidney disease (CKD) are lacking. Methods: This prospective observational cohort study enrolled 261 patients with CKD stages 1–5 and 30 subjects with normal renal function. Baseline serum zinc (Zn), selenium (Se), chromium, manganese, and copper, and laboratory tests were performed at enrolment. The primary endpoint was the presence of end-stage renal disease (ESRD) requiring long-term renal replacement therapy. Results: The median follow-up periods of renal and non-renal survivals were 67.78 and 29.03 months, respectively. Multiple linear regression showed that Zn and Se (β ± SE: 24.298 ± 8.616, *p* = 0.005; 60.316 ± 21.875, *p* = 0.006, respectively) levels were positively correlated with renal function. Time to ESRD was significantly longer for those with Zn levels ≥1287.24 ng/g and Se levels ≥189.28 ng/g (both *p* < 0.001). Cox regression analysis identified a higher Zn level as an independently negative predictor of ESRD after adjusting for renal function (hazard ratio, 0.450, *p* = 0.019). Conclusion: Serum Se and Zn concentrations are positively associated with renal function and better renal outcomes. A higher Zn concentration could independently predict better renal survival.

## 1. Introduction

The micronutrients also called trace elements, which include chromium (Cr), manganese (Mn), copper (Cu), selenium (Se) and zinc (Zn), are defined as minerals required by adults in amounts lower than 100 mg/day that are necessary for maintaining metabolism and life. Grave deficiency in these elements leads to severe illness or mortality [1,2,3,4,5]. Conversely, excess amounts put individuals at risk of metabolic disorders [6,7,8]. Several mechanisms may contribute to micronutrient deficiency in the milieu of chronic kidney disease (CKD), including leaching by dialysis, decreased intestinal absorption, altered gut microbiota, and uremic anorexia [9]. Serum Zn and Se levels in dialysis patients have been widely investigated because of their prevalent deficiency, ubiquitous biological roles in physiological function, and potential for antioxidation.

Zn deficiency is one of the most important micronutrient deficiencies globally and plays a crucial role in nutrition-related morbidity in developing countries. Severe Zn depletion results in growth failure, primary hypogonadism, impaired taste and smell, and compromised immunity [10,11,12]. Zn deficiency may be linked to several diseases such as malnutrition, diarrhea, malabsorption syndromes, inflammatory bowel disease, alcoholic cirrhosis, and sickle cell disease. Serum Zn levels were found to be lower in maintenance dialysis patients than in the general population; however, the exact mechanism remains unclear. Possible reasons include leaching during dialysis treatment sessions and altered gut microbiota [13,14]. Rashidi et al. and Lobo et al. demonstrated that Zn deficiency might increase oxidative stress and C-reactive protein levels, while a meta-analysis of randomized controlled trials indicated that Zn supplementation may improve the nutritional status of hemodialysis (HD) patients, resulting in higher dietary protein intake, higher superoxide dismutase levels, lower C-reactive protein levels, and lower malondialdehyde levels [15,16,17].

Se is present in multiple proteins as a constituent of various amino acids. Its best studied function is as a constituent of the antioxidant enzyme glutathione peroxidase (GSH-Px), although it also protects against the toxicity of heavy metals, such as mercury and lead [18]. Severe Se deficiency is associated with immune dysfunction, inflammatory bowel disease, and cardiovascular disease [19,20,21,22]. The serum Se concentration was found to be significantly lower in HD patients than in the general population. Additionally, Se deficiency may contribute to infection and perhaps uremic cardiomyopathy, thus leading to an increased risk of CVD in HD patients [23,24]. Serum Se levels are inversely associated with the risk of death among HD patients, and a prospective cohort study of Canadian HD patients even disclosed that lower serum Se levels were strongly and independently associated with mortality [25,26]. Oral Se supplementation improves the nutritional status of patients as well [27].

Previous studies concerning micronutrients mainly focused on the HD cohort, whereas the micronutrient status of non-dialysis CKD cohorts has rarely been explored. Meanwhile, the relationship between the concentrations of these micronutrients and renal outcomes also needs clarification. To fill this knowledge gap, we performed a prospective observational cohort study in non-dialysis CKD patients to further demarcate the association between the five micronutrients, demographics, and renal outcomes. These results may help optimize nutritional care strategies in patients with CKD.

## 2. Materials and Methods

### 2.1. Study Design and Patient Characteristics

This observational prospective cohort study assessed the baseline levels of micronutrients, including Zn, Se, Cr, Mn, and Cu, in a healthy population and CKD patients from the outpatient department of Chang Gung Memorial Hospital Keelung Branch between May 2013 and June 2020 and the Community Medicine Research Center between January 2017 and June 2020. A total of 30 healthy subjects and 261 non-dialysis CKD patients were enrolled in this study, and their renal function was classified into stage I to stage V according to the National Kidney Foundation Dialysis Outcomes Quality Initiative (NKF/DOQI) classification. All enrolled subjects participated in a multidisciplinary education (MPE) program based on the NKF/DOQI guidelines. The MPE program consisted of an integrated course of lectures on kidney protection, including nutrition, dietary principles, lifestyle modification, nephrotoxin refrainment, and pharmacological regimens delivered by well-trained case management nurses according to the guidelines in a standardized instruction booklet. The case management nurse contacted the patients to ensure a well-timed follow-up. Baseline demographics, clinical characteristics, and comorbidities were recorded, while micronutrient levels, biochemistry, and laboratory tests were estimated at enrolment into the study. The primary endpoint was end-stage renal disease requiring long-term renal replacement therapy (RRT). The criteria for RRT initiation were any estimated glomerular filtration rate (eGFR) meeting one of the following conditions and refractory to medical treatment: hyperkalemia, metabolic acidosis with a serum bicarbonate concentration of <13 mEq/L, fluid overload due to oliguria, or severe uremic syndrome (uremic bleeding, pericarditis, encephalopathy, pruritus, or vomiting).

This study was performed in accordance with the Declaration of Helsinki and approved by the Ethics Committee of the Institutional Review Board at Chang Gung Memorial Hospital (IRB:201800275B0C602 and 201900188B0C502). Written informed consent was obtained from all participants.

### 2.2. Sample Collection

Blood samples were collected after overnight fasting and delivered immediately (within 4 h of collection) to the laboratory for biochemical analyses and micronutrient measurements. A fraction of the samples was transferred to chilled tubes and centrifuged at 3000× *g* for 10 min at 4 °C to obtain sera. Lipemic or hemolyzed serum samples were discarded.

### 2.3. Chemicals and Reagents for Micronutrients Measurement

Nitric acid was obtained from Honeywell Fluka (≥65%; Seelze, Germany). Cu, Cr, Mn, and Se were obtained from Merck (1000 µg/mL ICP Standard; Darmstadt, Germany). Zn and Rhodium (Rh) were purchased from AccuStandard (1000 µg/mL ICP Standard, New Haven, CT, USA). Ultrapure water was prepared using the Direct-Q® 3 UV water purification system (Merck, Darmstadt, Germany). The Rh standard solution was further diluted to 10 µg/mL with 13% HNO3 as an internal standard (IS) solution for sample preparation.

### 2.4. Sample Preparation

Human sera (0.2 g) were added to 5 µL of IS solution (10 µg/mL Rh) and 1 mL of nitric acid in 15 mL centrifuge tubes (polypropylene conical tube, Falcon, Tamaulipas, Mexico). The samples were transferred to a microwave digestion system (MARS 6, CEM, Matthews, NC, USA), which was equipped with a fiber optic temperature sensor and a high-throughput accessory (CEM High Throughput Vessel set, DV-120 turntable, Matthews, NC, USA). The temperature was ramped up for 30 min and held at 100 °C for 20 min with the oven power set at 750 W. After this process, the samples were cooled to room temperature, and the final volume was turned up to 5 mL with ultrapure water. The samples were centrifuged at 4000 rpm for 5 min, and the supernatant was introduced directly into an inductively coupled plasma mass spectrometry (ICP-MS, PerkinElmer NexIon 350X, Waltham, MA, USA) system to analyze the elements present. In addition, 0.2 g of human sera was added to the IS solution, and the 5-element solution was added to the spiked sample. The final concentrations were 15 ppb for copper and Zn, and 3 ppb for Cr, Mn, and Se in the instrumental analysis. Spike samples were used to calculate the recovery and confirm the accuracy of this method.

### 2.5. Inductively Coupled Plasma Mass Spectrometry (ICP-MS) Analysis

Humoral Cu, Zn, Cr, Mn, and Se concentrations were determined by ICP-MS. The standard solution was diluted to the concentration levels determined using calibration curves (including a 10-ng/mL internal standard) with 13% HNO3. The ranges of the calibration curves were 0.005–50 ng/mL for Cu, Zn, Cr, Mn, and Se. ICP-MS analysis was performed with a nickel cone and glass cyclonic spray chamber. The instrumental settings and conditions were as follows: RF power was set at 1500 W; and plasma, auxiliary, and nebulizer gas flow rates were set at 18.0, 1.20, and 0.92 L/min, respectively. The instrument was operated using methane as the collision cell gas to remove interference. Copper and Zn were detected in standard mode, whereas Cr, Mn, and Se were detected in dynamic reaction cell (DRC) mode. The measured analyte masses/IS masses were Cu^65^/Rh^103^, Zn^67^/Rh^103^, Cr^52^/Rh^103^, Mn^55^/Rh^103^, and Se^78^/Rh^103^. Instrument control, data acquisition, and evaluation were performed using the Syngistix software (PerkinElmer, Waltham, MA, USA). The optimized conditions are presented in Table S1. The contents of the five elements were expressed in human sera (ng/g).

### 2.6. Statistical Analysis

Continuous variables were tested for normal distribution using skewness, kurtosis, and the Kolmogorov–Smirnov test. Normally distributed variables, whose values were expressed as means (standard deviations), were compared using one-way analysis of variance (ANOVA), while categorical variables were tested using the Chi-squared test. The non-parametric independent Kruskal–Wallis test was performed to compare non-normally distributed variables expressed as medians (interquartile ranges).

Simple linear regression was applied to examine the association between independent variables and eGFR. Non-normally distributed variables were logarithmically transformed as appropriate. Multiple regression was used to adjust for age and sex (Model 1) or all confounding factors (Model 2). Univariate analysis followed by multivariate logistic regression analysis (enter method) was applied after adjusting for potential confounders, such as age and sex (Model 1) and all other variables (Model 2) to identify the odds ratio of clinical variables associated with the primary endpoint.

Receiver operating characteristic (ROC) curves were plotted to predict the probability of a binary outcome, including Se, Zn, and a combination of Se and Zn vs. the primary endpoint. Differences were examined using the area under the ROC curve (AUC). The cut-off values of Se and Zn concentrations were obtained from the ROC curve using Youden’s index. Analysis of the time to the primary endpoint was derived from Kaplan–Meier analysis. Univariate analysis followed by multivariable Cox regression analysis was used to assess the hazard ratio of clinical variables associated with the primary endpoint after adjusting for age and sex (Model 1), adjusting for all variables, except eGFR (Model 2), and all other variables (Model 3).

**Figure 1:**
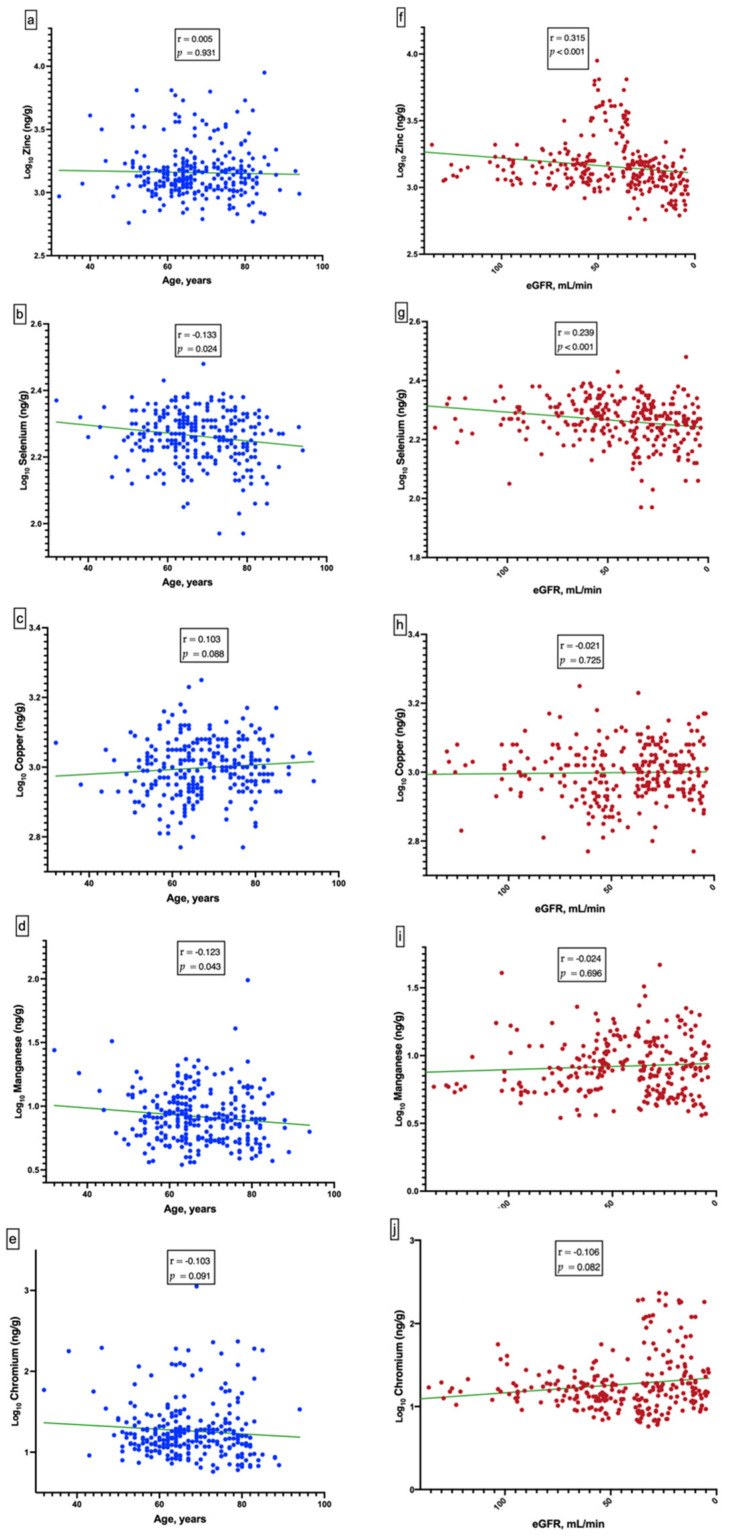
Correlations between age and logarithmic transformed trace elements [age vs. Zn (Panel (**a**)); age vs. Se (Panel (**b**)); age vs. Cu (Panel (**c**)); age vs. Mn (Panel (**d**)); age vs. Cr (Panel (**e**)). Correlations between estimated glomerular filtration rate (eGFR) and logarithmic transformed trace elements [eGFR vs. Zn (Panel (**f**)); eGFR vs. Se (Panel (**g**)); eGFR vs. Cu (Panel (**h**)); eGFR vs. Mn (Panel (**i**)); eGFR vs. Cr (Panel (**j**))].

**Figure 2:**
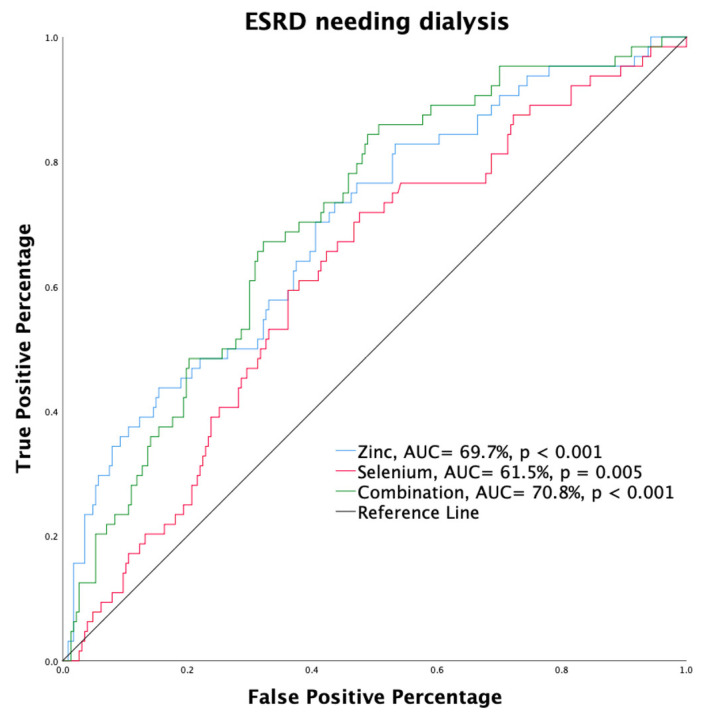
A receiver operating characteristic curve illustrating the performance of low zinc (AUC: 69.7%, *p* < 0.001) and selenium (AUC: 61.5%, *p* = 0.005) levels and their combination, obtained by multiplying both values, (AUC: 70.8%, *p* < 0.001) in predicting the development of end-stage renal disease.

**Figure 3:**
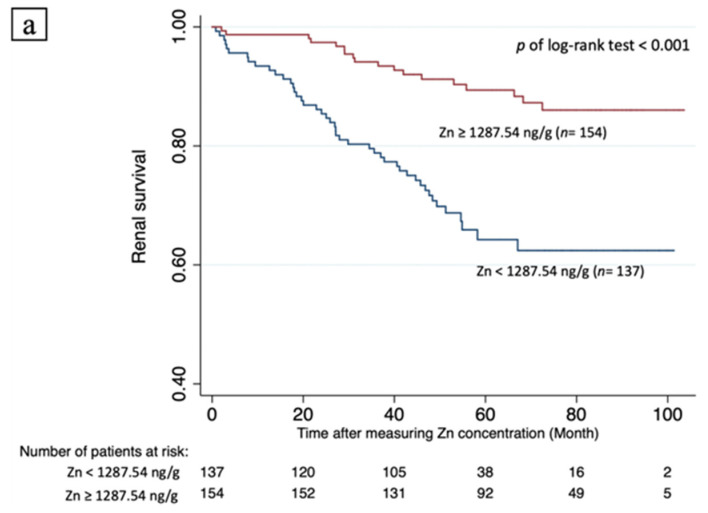

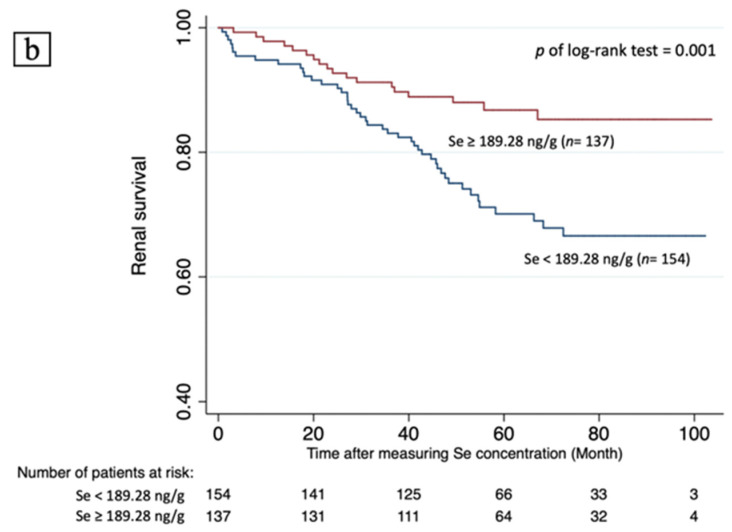
Kaplan–Meier survival curves in all participants according to serum zinc and selenium concentration. Renal survival was significantly better for individuals with Zn levels ≥1287.24 ng/g (n = 137) than for those with Zn levels <1287.24 ng/g (n = 154) (Cox–Mantel log rank test, *p* < 0.001) (Panel (**a**)). Individuals with Se levels ≥189.28 ng/g (n = 154) had significantly better renal survival than those with Se levels <189.28 ng/g (n = 137) (Cox–Mantel log rank test, *p* = 0.001) (Panel (**b**)).

Pearson or Spearman correlation coefficients were used to test the correlation between Zn, Se, Cu, Mn, and Cr levels and age or eGFR. All statistical analyses were two-tailed, and a value of *p* < 0.05 was considered statistically significant. Data were analyzed using the Statistical Package for the Social Sciences (SPSS, Inc., Chicago, IL, USA) version 26.0 for Mac. GraphPad Prism version 9 (GraphPad Software, Inc., San Diego, CA, USA) was used to generate the graph in Figure 1, SPSS was used to generate the graph in Figure 2, and Stata/MP 14.1 for Mac (StataCorp LLC, College Station, TX, USA) was used to generate the graph shown in Figure 3.

## 3. Results

### 3.1. Study Design and Subject Characteristics

This prospective observational study was based on data from the MPE program staged by the CKD center of the Chang Gung Memorial Hospital Keelung Branch between May 2013 and June 2020. A total of 30 healthy individuals and 261 patients with CKD were enrolled in this study. The CKD cohort comprised of 2, 44, 107, 65, and 43 individuals with CKD ranging from stages 1 to 5, respectively. All participants were screened for underlying diseases and demographics through interviews and electronic medical records. Baseline serum levels of micronutrients and laboratory test results were estimated at enrollment. All participants underwent the MPE program and regular outpatient department follow-up. The median follow-up period of renal survivals was 67.78 (49.45–83.90) months and non-renal survivals was 29.03 (17.80–45.73) months (*p* < 0.001). The non-renal survivals had lower eGFR (20.09 ± 15.53 vs. 51.17 ± 28.39 mL/min/1.73 m2, *p* < 0.001), lower albumin level (4.09 ± 0.42 vs. 4.34 ± 0.43 g/dL, *p* < 0.001), lower hemoglobin level (10.52 ± 1.58 vs. 12.37 ± 1.95 g/dL, *p* < 0.001) and higher phosphorus level (4.52 ± 1.13 vs. 3.84 ± 0.65 mg/dL, *p* < 0.001). Serum Zn and Se levels were significantly higher in the renal survivals than non-renal survivals (1416.91 vs. 1169.17 ng/g, *p* < 0.001; 190.45 vs. 176.40 ng/g, *p* = 0.005, respectively) (Table 1). The association between serum levels of micronutrients, age, and eGFR was analyzed. Pearson’s correlation coefficients (r) between serum Se/Mn and age were −0.133 (*p* = 0.024) and −0.123 (*p* = 0.043), respectively. The r between serum Zn/Se and eGFR were 0.315 (*p* < 0.001) and 0.239 (*p* < 0.001), respectively. This suggests that Zn and Se levels declined significantly with kidney function deterioration (Figure 1).

### 3.2. Demographics and Clinical Characteristics Comparisons among Various CKD Stages

As indicated by one-way ANOVA, serum Zn and Se levels appeared to be distributed in a descending manner from CKD stage 0 to stage 5 (*p* trend < 0.001 and *p* trend = 0.001, respectively), while the serum Cr level was positively associated with a decline in eGFR (*p* trend = 0.013) (Table 2). Albumin, hemoglobin, and calcium levels also appeared to be distributed in a descending manner (*p* trend < 0.001), and the phosphorus level was positively associated with CKD progression (*p* trend < 0.001). Moreover, the percentage of patients with hypertension increased as CKD progressed (*p* trend = 0.007) (Table 2).

### 3.3. Kidney Function and Independent Variables

For the entire cohort, simple linear regression for eGFR and independent variables revealed that Zn (β ± SE: 22.139 ± 8.692, *p* = 0.004), Se (β ± SE: 77.571 ± 22.031, *p* = 0.001), and albumin (β ± SE: 16.474 ± 3.333, *p* < 0.001) levels were positively associated with eGFR, and that the Cr (β ± SE: −13.131 ± 5.122, *p* = 0.011) level was inversely associated with eGFR (Table 3). Multiple linear regression analysis with age and sex adjustment (Model 1) also showed similar results: Zn, Se, and albumin (β ± SE: 24.298 ± 8.616, *p* = 0.005; 60.316 ± 21.875, *p* = 0.006; 19.824 ± 3.703, *p* < 0.001, respectively) level were significantly positively linked to kidney function. However, multiple regression with adjustment for all variables (Model 2) showed that only the albumin level was associated with eGFR (Table 3).

### 3.4. Relationship of Renal Outcome and Independent Variables

Through univariate logistic regression analysis (crude), we identified Zn, Se, and albumin levels as predictors of end-stage renal disease (ESRD) requiring dialysis (OR, 0.999, 95% CI: 0.998–1.000, *p* = 0.002; OR, 0.991, 95% CI: 0.982–1.000, *p* = 0.043; OR, 0.290, 95% CI: 0.152–0.553, *p* < 0.001, respectively) (Table 4). After multiple logistic regression with age and sex adjustment (Model 1), low serum levels of Zn, Se, and albumin (OR, 0.999, 95% CI: 0.998–1.000, *p* = 0.002; OR, 0.990, 95% CI: 0.981–0.999, *p* = 0.042; OR, 0.255, 95% CI: 0.130–0.502, *p* < 0.001, respectively) predicted ESRD. Through multiple logistic regression with adjustment for age, sex, and other continuous variables (Model 2), Zn and albumin (OR, 0.999, 95% CI: 0.998–1.000, *p* = 0.027 and OR, 0.381, 95% CI: 0.174–0.833, *p* = 0.016) levels could serve as independent factors associated with ESRD (Table 4). The association of Zn and Se with ESRD was consistent when assessed in a categorical fashion using univariate analysis followed by multiple logistic regression analyses. The middle-third serum Zn concentration subgroup had a higher risk of ESRD than the upper-third subgroup (crude: OR, 5.000, 95% CI: 2.233–11.194, *p* < 0.001; Model 1: OR, 5.012, 95% CI: 2.211–11.359, *p* < 0.001; Model 2: OR, 4.105, 95% CI: 1.697–9.930, *p* = 0.002). The lower-third serum Zn concentration subgroup also had a higher risk of ESRD than the upper-third subgroup (crude: OR, 2.793; 95% CI: 1.291–6.847, *p* = 0.01; Model 1: OR, 2.987, 95% CI: 1.291–6.912, *p* = 0.011; Model 2: OR, 2.808, 95% CI: 1.161–6.789, *p* = 0.022) (Table 4). For Se, only the middle-third subgroup (crude and Model 1) had a significantly higher risk of ESRD than the upper-third subgroup (OR, 2.306; 95% CI: 1.141–4.662, *p* = 0.018; OR, 2.331; 95% CI: 1.142–4.759; *p* = 0.020, respectively). The associations of low serum albumin level and diabetes with ESRD were also significantly positive (crude, Model 1, and Model 2) after adjusting for all variables in a categorical manner (Table 4). Figure 2 shows the ROC curve illustrating the ability of low Zn levels (AUC: 69.7%, *p* < 0.001), low Se levels (AUC: 61.5%, *p* = 0.005), and their combination, obtained by multiplying the two, (AUC: 70.8%, *p* < 0.001) in predicting ESRD development. These results suggest that there might be an interaction between Zn and Se that affects eGFR.

### 3.5. Renal Survival and Predicting Factors

Figure 3 illustrates the Kaplan–Meier survival curves for all participants according to serum Zn and Se concentrations. Renal survival was significantly better for individuals with Zn levels ≥1287.24 ng/g (n = 137) than those with Zn levels <1287.24 ng/g (n = 154) (Cox–Mantel log rank test, *p* < 0.001). Individuals with Se levels ≥189.28 ng/g (n = 154) had significantly better renal survival than those with Se levels <189.28 ng/g (n = 137) (Cox–Mantel log rank test, *p* = 0.001). Table 5 shows the hazard ratios (HR) for renal progression to ESRD. Cox regression analysis identified serum Zn level ≥1287.24 ng/g as an independently negative predictor of ESRD, independent of eGFR (crude: HR, 0.263, 95% CI: 0.152–0.455, *p* < 0.001; Model 1: HR, 0.259, 95% CI: 0.148–0.454, *p* < 0.001; Model 2: HR, 0.241, 95% CI: 0.128–0.453, *p* < 0.001; Model 3: HR, 0.450, 95% CI: 0.231–0.878, *p* = 0.019) (Table 5). Except in Model 3, a serum Se level ≥189.28 ng/g was a negative predictor of ESRD through Cox regression analysis, (crude: HR, 0.472, 95% CI: 0.273–0.816, *p* = 0.007; Model 1: HR, 0.454, 95% CI: 0.260–0.795, *p* = 0.006; Model 2: HR, 0.473, 95% CI: 0.258–0.866, *p* = 0.015; Model 3: HR, 0.641, 95% CI: 0.346–1.187, *p* = 0.157). Meanwhile, eGFR served as a significant independent predictor of ESRD in univariate and all models of multivariable Cox analyses (crude: HR, 0.927, 95% CI: 0.908–0.946, *p* < 0.001; Model 1: HR, 0.919, 95% CI: 0.899–0.940, *p* < 0.001; Model 3: HR, 0.921, 95% CI:0.897–0.946, *p* < 0.001) (Table 5).

## 4. Discussion

In this prospective observational cohort study, we demonstrated that serum concentrations of Zn and Se were associated with kidney function, and that Zn levels could significantly predict renal survival, independent of albumin, eGFR, age, sex, and other comorbidities. In general, serum Zn and Se levels gradually decreased along with the decline in eGFR, and higher serum Zn and Se concentrations are associated with better renal survival, especially for Zn. Overall, the results indicate that the two micronutrients might play crucial roles in the progression of CKD.

Micronutrients in humans are responsible for numerous important physiological effects. In particular, Zn, Se, and Mn play crucial roles in maintaining health. Zn is involved in various biochemical pathways as a cofactor for over 300 enzymes. It has been proven that Zn deficiency results in many morbidities, such as male reproductive disturbance, impaired immunity, loss of appetite, and susceptibility to coronavirus infection disease 2019 [12,28,29,30,31]. The principal effect of Se is antioxidation as a constituent of the antioxidant enzyme GSH-Px, which protects humans from heavy metal toxicity, eliminates free radicals, and maintains adequate immunity [18]. Se deficiency is linked to immune dysfunction and cardiovascular disease [22,30]. Therefore, ensuring adequate nutritional intake and maintaining homeostasis of both micronutrients is important.

Micronutrient alterations in HD have been reported previously. Cadmium, Cr, nickel, vanadium, copper, and lead accumulated, whereas essential elements such as Zn, Se, and Mn were deficient in the HD cohort [32]. This phenomenon may be attributed to essential leaching into the dialysate during HD and gut microbiota alteration, leading to malabsorption. However, few studies have investigated the status of micronutrients in non-dialysis CKD patients. To the best of our knowledge, this study is the first to explore this issue with the longest observation time and comprising subjects from all stages of CKD. We found that when CKD progressed, Zn and Se levels decreased, whereas Cr levels tended to increase; these findings are similar to those in the HD cohort. Our participants mainly originated from the PME program, which encourages consumption of CKD diets, (low protein, low sodium, low potassium, and low phosphorus), which might affect micronutrient intake. As expected, serum albumin levels gradually decreased in patients with advanced CKD and had an independent linear relationship with eGFR, indirectly revealing that protein–energy wasting syndrome was prevalent in patients with late CKD [33]. The malnutrition status may have affected the absorption of micronutrients to a certain extent in the CKD cohort.

Risk factors that accelerate renal function include cigarette smoking, uncontrolled hypertension, diabetes, gender, malnutrition, and old age [34,35]. However, few studies have explored the relationship between Zn levels and CKD progression. Atsuyuki Tokuyama et al. found that Zn deficiency is a risk factor for ESRD among advanced CKD individuals over a one-year observation period [36]. Damianaki et al. found a significant association between a lower baseline Zn level and deterioration of kidney function, but it was no longer significant after baseline eGFR or proteinuria was introduced into this model [37]. Higher serum Se levels were inversely associated with all-cause and infection-related deaths, and lower Se intake was associated with higher CKD prevalence [25,38]. Cox regression analyses using univariate and multivariable models adjusted for age, sex, and diabetes revealed that high Zn and Se levels were significant predictive factors of ESRD. Se was no longer a significant predictor after introducing eGFR, whereas Zn was still an independent predictor of ESRD. The median time to ESRD of non-renal survivals was 29.03 months, and the median tracking time of renal survivals was 67.78 months. These results suggest that serum Zn levels might play a crucial role in CKD progression. Further large-scale longitudinal studies are required to consolidate this finding. Besides, Zn levels vary widely among individuals and are highly influenced by nutritional status, geographic location of residence, and other comorbidities. In order to alleviate these confounding factors, the participants were majorly recruited from northern Taiwan; Zn and renal outcomes were analyzed through adjustment for age, gender, albumin, eGFR, cardiovascular disease and other comorbidities. 

Oxidative stress plays a crucial role in various diseases, including CKD [39]. Antioxidant efficiency diminished with renal deterioration and reached a nadir in ESRD patients [40]. GSH-Px, mainly produced in the proximal renal tubules, requires Se as a cofactor and is an important cellular component in preventing chronic inflammation and protecting against damage caused by reactive oxygen species (ROS) [18,41]. Although Se is generally viewed as an antioxidant nutrient, it has no antioxidant capability and is only a prerequisite for the synthesis and activity of several antioxidant enzymes [42]. Se and plasma GSH-Px were both significantly reduced in accordance with a decline in eGFR; however, Se supplementation could increase blood GSH-Px activity in the CKD cohort and has been demonstrated to significantly improve renal function when co-administered with coenzyme Q10 in Se-deficient elderly patients [43,44,45]. Zn plays a key role in antioxidation as it has been found to mitigate ROS and is also imperative in the active site of superoxide dismutase that catalyzes the dismutation of superoxide [46,47]. An in vitro study indicated that in diabetic human renal tubular cells, Zn treatment augmented the expression of nuclear factor erythroid 2-related factor 2 (Nrf2), a master regulator of genes linked to antioxidant effects and detoxification [48,49]. Pedruzzi et al. demonstrated that HD patients have downregulated Nrf2 mRNA expression [50]. Both Zn and Se have specific antioxidant capacity, and oxidative stress plays an aggravating role in the process of renal fibrosis, which partly explains why higher baseline serum Zn and Se levels predict better renal outcomes [51].

A systematic review and meta-analysis by Wang et al. demonstrated that Zn supplementation may benefit nutrition in HD patients, including lowering C-reactive protein and malondialdehyde levels [17]. A randomized controlled study by Escobedo-Monge also showed that Zn supplementation exerts a small but significant nutritional improvement in children and adolescents with CKD [52]; therefore, Se dose supplementation alleviated oxidative stress and inflammation in HD patients [27,52]. Zn may protect against phosphate-induced calcification in CKD and may be associated with a lower risk of abdominal aorta calcification and a lower risk of cardiovascular disease in the CKD cohort [1,53,54]. The current findings suggest that supplementation benefits nutritional status and alleviates oxidative stress in the CKD cohort. Nevertheless, whether Zn or Se supplementation improves overall survival or renal survival in non-dialysis CKD still requires additional evidence. It may be more appropriate to estimate serum Zn and Se levels prior to supplementation.

This study has some limitations. First, as a prospective observational study, we only estimated baseline micronutrients, demographics, and other biochemical data at enrollment and analyzed the results. We did not additionally analyze the interim data. Therefore, it was not possible to determine whether fluctuations in data during the observation period affected the results. Second, the time points at which participants were recruited into the study were highly heterogeneous, and the follow-up duration of renal survival ranged from 2 to 8 years because the participants were primarily from the PME program (since 2011) and Community Medicine Research Center (since 2017). Third, dietary habits and lifestyle were not analyzed for factors possibly influencing micronutrient intake and absorption. Fourth, relatively few subjects were enrolled in the study. However, the number of subjects enrolled in our study was already the largest among the relevant studies on micronutrients in CKD, and the tracking time was also the longest; these factors probably increase the certainty of the obtained results.

## 5. Conclusions

This prospective observational cohort study established associations between micronutrients and renal outcomes. Serum Zn and Se concentrations were positively associated with eGFR, while higher Zn and Se concentrations were linked to better renal survival after adjustment for age, sex, diabetes, albumin, and other comorbidities. Specifically, higher serum Zn levels significantly predicted better renal survival independent of eGFR and all other above-mentioned variables. The results suggest that monitoring micronutrients in patients with CKD is necessary, and timely supplementation and adequate education of dietary habits might be beneficial for renal outcomes.

## Figures and Tables

**Table 1:** Demographics and clinical characteristics of renal survivals and non-renal survivals.

	Renal Survival, n = 227	Non-Renal Survival, n = 64	*p* Value
Age, year	67.25 ± 10.23	67.47 ± 10.30	0.878
Observation period, month	67.78 (49.45–83.90)	29.03 (17.80–45.73)	< 0.001 *
Male, n (%)	117 (51.5)	31 (48.4)	0.661
Creatinine, mg/dL	1.73 ± 1.24	4.16 ± 2.40	< 0.001 *
eGFR, mL/min/1.73 m^2^	51.17 ± 28.39	20.09 ± 15.53	< 0.001 *
Albumin, g/dL	4.34 ± 0.43	4.09 ± 0.42	< 0.001 *
Hemoglobin, g/dL	12.37 ± 1.95	10.52 ± 1.58	< 0.001 *
Ca, mg/dL	9.27 ± 0.60	8.98 ± 0.58	0.001
P, mg/dL	3.84 ± 0.65	4.52 ± 1.13	< 0.001 *
Body mass index	26.06 ± 4.19	25.82 ± 5.17	0.707
Zinc, ng/g ^$^	1416.91 (1145.87–1701.87)	1169.17 (943.05–1380.41)	< 0.001 ^&^*
Selenium, ng/g ^$^	190.45 (170.76–209.61)	176.40 (161.81–193.15)	0.005 ^&^*
Copper, ng/g^$^	996.19 (883.11–1120.38)	1017.17 (880.04–1154.45)	0.680 ^&^
Manganese, ng/g ^$^	7.99 (5.77–11.72)	7.97 (5.11–9.76)	0.174 ^&^
Chromium, ng/g^$^	15.09 (11.45–21.22)	16.84 (12.18–30.12)	0.074 ^&^
Diabetes, n (%)	104 (45.8)	40 (62.5)	0.023 *
Hypertension, n (%)	185 (81.5)	57 (89.0)	0.213
ASCVD, n (%)	65 (28.6)	18 (28.1)	0.897
CAD, n (%)	42 (18.5)	13 (20.3)	0.761
CHF, n (%)	21 (9.3)	3 (4.7)	0.236
CVA, n (%)	8 (3.5)	2 (3.1)	1.000
PAD, n (%)	3 (1.3)	1 (1.6)	1.000

Notes: Data are presented as mean ± standard deviation or median (interquartile range); Abbreviations: CKD, chronic kidney disease; eGFR, estimated glomerular filtration rate; ASCVD, atherosclerotic cardiovascular disease; CAD, coronary artery disease; CHF, congestive heart failure; CVA, cerebrovascular disease; PAD, peripheral artery disease; *: Statistically significant; ^$^: non-normally distributed; ^&^: Comparison between renal survivals and non-renal survivals using Mann–Whitney U-test.

**Table 2:** Demographics and clinical characteristics among various CKD stages.

	Stage 0–1 (n = 32)	Stage 2 (n = 44)	Stage 3 (n = 107)	Stage 4 (n = 65)	Stage 5 (n = 43)	*p* for Trend
Age, year	61.25 ± 7.55	65.53 ± 7.44	67.47 ± 10.80	70.31 ± 11.12	68.63 ± 9.69	<0.001 ^&,^*
Male, n (%)	13 (40.6)	24 (54.5)	63 (58.9)	32 (49.2)	16 (37.2)	0.434 ^@^
Creatinine, mg/dL	0.68 ± 0.15	1.00 ± 0.19	1.56 ± 0.32	2.61 ± 0.69	5.94 ± 1.99	<0.001 ^&,^*
eGFR, mL/min/1.73 m^2^	103.81 ± 19.85	68.34 ± 8.32	43.66 ± 9.46	23.84 ± 6.38	9.06 ± 3.20	<0.001 ^&,^*
Albumin, g/dL	4.56 ± 0.22	4.43 ± 0.38	4.35 ± 0.38	4.14 ± 0.44	4.03 ± 0.55	<0.001 ^&,^*
Hemoglobin, g/dL	13.37 ± 1.11	13.50 ± 1.64	12.55 ± 1.66	10.89 ± 1.51	9.57 ± 1.29	<0.001 ^&,^*
Calcium, mg/dL	9.30 ± 0.37	9.34 ± 0.36	9.35 ± 0.41	9.18 ± 0.84	8.67 ± 0.60	<0.001 ^&,^*
Phosphorus, mg/dL	3.74 ± 0.52	3.73 ± 0.48	3.79 ± 0.59	3.95 ± 0.67	4.98 ± 1.19	<0.001 ^&,^*
Body mass index	25.15 ± 2.87	26.42 ± 4.11	26.93 ± 4.81	25.04 ± 3.61	25.34 ± 5.30	0.354 ^&^
Zinc, ng/g ^$^	1387.14 (1159.11–1670.47)	1433 (1173.62–1600.39)	1511.59 (1156.25–3104.54)	1285.86 (1093.10–1497.03)	1015.07 (791.61–1191.06)	<0.001 ^$,^*
Selenium, ng/g ^$^	187.96 (175.75–206.95)	196.07 (176.54–213.11)	193.40 (169.88–216.15)	177.66 (159.81–194.75)	177.28 (150.98–192.67)	0.001 ^$,^*
Copper, ng/g ^$^	1025.16 (967.95–1092.79)	994.27 (846.29–1133.45)	957.15 (851.16–1120.62)	1024.51 (931.86–1125.91)	973.56 (859.99–1191.79)	0.817 ^$^
Manganese, ng/g ^$^	5.96 (5.52–7.65)	7.42 (5.49–9.48)	8.72 (7.37–12.59)	7.46 (5.14–11.85)	8.24 (5.18–10.31)	0.376 ^$^
Chromium, ng/g ^$^	15.60 (12.87–19.37)	16.44 (11.87–19.19)	12.53 (9.14–19.32)	18.62 (12.50–40.60)	15.40 (12.43–26.34)	0.013 ^$,^*
Diabetes, n (%)	18 (56.3)	18 (40.9)	53 (49.5)	33 (50.8)	22 (51.2)	0.881 ^@^
Hypertension, n (%)	20 (62.5)	38 (86.4)	90 (84.1)	54 (83.1)	40 (93)	0.007 ^@,^*
ASCVD, n (%)	5 (15.6)	12 (27.3)	35 (32.7)	23 (35.4)	8 (18.6)	0.648 ^@^
CAD, n (%)	2 (6.3)	7 (15.9)	22 (20.6)	19 (29.2)	5 (11.6)	0.211 ^@^
CHF, n (%)	2 (6.3)	3 (6.8)	7 (6.5))	10 (15.4)	2 (4.7)	0.534 ^@^
CVA, n (%)	0 (0)	2 (4.5)	5 (4.7)	3 (4.6)	0 (0)	0.898 ^@^
PAD, n (%)	1 (3.1)	0 (0)	2 (1.9)	0 (0)	1 (3.1)	0.802 ^@^

Notes: Data are presented as mean ± standard deviation or median (interquartile range); Abbreviations: CKD, chronic kidney disease; eGFR, estimated glomerular filtration rate; ASCVD, atherosclerotic cardiovascular disease; CAD, coronary artery disease; CHF, congestive heart failure; CVA, cerebrovascular disease; PAD, peripheral artery disease; *: Statistically significant; ^$^: Variable was log_10_ transformed and compared among subjects with different CKD stages by one-way analysis of variance (ANOVA) with linear trend; ^&^: Comparison among subjects with different CKD stages by one-way ANOVA with linear trend; ^@^: Comparison among subjects with different CKD stages by Chi-square test via linear-by-linear association.

**Table 3:** β-coefficient between eGFR and independent variables.

	Simple Linear Regression		Multiple Regression Analysis, Model 1		Multiple Regression Analysis, Model 2	
	β ± SE	*p*	β ± SE	*p*	β ± SE	*p*
Age	−0.769 ± 0.163	<0.001 *	-	-	−0.591 ± 0.178	0.001 *
Gender ^&^	1.382 ± 3.461	0.690	-	-	−0.161 ± 3.572	0.964
Diabetes ^&^	−1.093 ± 3.459	0.752	−1.069 ± 3.357	0.750	−2.954 ± 3.469	0.395
Albumin	16.474 ± 3.333	<0.001 *	19.824 ± 3.703	<0.001 *	17.554 ± 4.228	<0.001 *
ASCVD ^&^	−5.489 ± 3.798	0.150	−2.719 ± 3.739	0.468	−3.480 ± 3.782	0.359
Body mass index	0.195 ± 0.395	0.622	0.075 ± 0.385	0.847	0.043 ± 0.391	0.912
Chromium ^$^	−13.131 ± 5.122	0.011 *	−15.218 ± 5.011	0.003 *	−9.380 ± 5.782	0.106
Manganese ^$^	−9.305 ± 8.631	0.282	−14.604 ± 8.605	0.091	−16.034 ± 9.953	0.108
Copper ^$^	−7.661 ± 22.862	0.738	1.408 ± 23.135	0.952	0.744 ± 22.280	0.973
Zinc ^$^	25.139 ± 8.692	0.004 *	24.298 ± 8.616	0.005 *	16.959 ± 10.982	0.124
Selenium ^$^	77.571 ± 22.031	0.001 *	60.316 ± 21.875	0.006 *	23.257 ± 23.220	0.318

Enter selection method was applied for multiple linear regression analysis. Abbreviations: ASCVD, atherosclerotic cardiovascular disease, including coronary artery disease, stroke, transient ischemic attack, or peripheral arterial disease from atherosclerosis; eGFR, estimated glomerular filtration rate *: Statistically significant, ^$^: Log_10_ transformed, ^&^: categorical variable; Model 1: adjusted for age and gender; Model 2: adjusted for age, gender, and all other variables.

**Table 4:** OR of variables associated with ESRD needing dialysis.

	Crude		Model 1		Model 2	
Variable	OR (95% CI)	*p* Value	OR (95% CI)	*p* Value	OR (95% CI)	*p* Value
Continuous variables						
Zn, ng/g	0.999 (0.998–1.000)	0.002 *	0.999 (0.998–1.000)	0.002 *	0.999 (0.998–1.000) ^&^	0.027 *
Se, ng/g	0.991 (0.982–1.000)	0.043 *	0.990 (0.981–0.999)	0.042 *	0.998 (0.988–1.009) ^&^	0.729
Cu, ng/g	1.000 (0.999 –1.002)	0.810	1.000 (0.999–1.002)	0.847	1.000 (0.999–1.002) ^&^	0.738
Mn, ng/g	0.967 (0.911–1.027)	0.276	0.973 (0.916–1.033)	0.371	0.955 (0.881–1.035) ^&^	0.261
Cr, ng/g	1.005 (0.999–1.011)	0.113	1.005 (0.999–1.012)	0.102	1.008 (0.999–1.017) ^&^	0.083
Albumin, g/dL	0.290 (0.152–0.553)	<0.001 *	0.255 (0.130–0.502)	<0.001 *	0.381 (0.174–0.833) ^&^	0.016 *
Body mass index, kg/m^2^	0.991 (0.930–1.057)	0.794	0.990 (0.928–1.057)	0.766	1.012 (0.944–0.084) ^&^	0.741
Categorical variables						
Zn > 1511.5396 ng/g	1		1		1	
Zn = 1167.5252–1511.5396 ng/g	5.000 (2.233–11.194)	<0.001 *	5.012 (2.211–11.359)	<0.001 *	4.105 (1.697–9.930) ^#^	0.002 *
Zn < 1167.5252 ng/g	2.973 (1.291–6.847)	0.010 *	2.987 (1.291–6.912)	0.011 *	2.808 (1.161–6.788) ^#^	0.022 *
Se > 198.454 ng/g	1		1		1	
Se = 174.2536–198.4540 ng/g	2.306 (1.141–4.662)	0.018 *	2.331 (1.142–4.759)	0.020 *	1.396 (0.637–3.060) ^#^	0.405
Se < 174.2536 ng/g	1.474 (0.705–3.082)	0.303	1.445 (0.690–3.026)	0.329	1.388 (0.623–3.092) ^#^	0.423
Albumin < 4 g/dL	3.930 (2.108–7.327)	<0.001 *	4.257 (2.243–8.077)	<0.001 *	3.751 (1.861–7.561) ^#^	<0.001 *
ASCVD (yes vs. no)	0.893 (0.477–1.673)	0.724	0.903 (0.477–1.708)	0.753	0.843 (0.407–1.745) ^#^	0.646
Diabetes (yes vs. no)	1.857 (1.048–3.290)	<0.001 *	1.848 (1.040–3.282)	0.036 *	2.525 (1.308–4.875) ^#^	0.006 *

Abbreviations: OR, odd ratios; CI, confidence interval; ASCVD, atherosclerotic cardiovascular disease; ESRD, end-stage renal disease. Model 1: adjusted for age and gender. Model 2: ^&^, adjusted for age, gender, and all other continuous variables; ^#^, adjusted for age, gender, and all other categorical variables. ^*^: statistically significant

**Table 5:** Cox regression analysis on the association of factors and ESRD needing dialysis.

	Univariate, Crude		Multivariable, Model 1		Multivariable, Model 2		Multivariable, Model 3	
	HR (95% CI)	*p* Value	HR (95% CI)	*p* Value	HR (95% CI)	*p* Value	HR (95% CI)	*p* Value
Zn ≥ 1287.24 ng/g ^#^	0.263 (0.152–0.455)	<0.001 *	0.259 (0.148–0.454)	<0.001 *	0.241 (0.128–0.453)	<0.001 *	0.450 (0.231–0.878)	0.019 *
Se ≥ 189.28 ng/g ^#^	0.472 (0.273–0.816)	0.007 *	0.454 (0.260–0.795)	0.006 *	0.473 (0.258–0.866)	0.015 *	0.641 (0.346–1.187)	0.157
Male	0.802 (0.488–1.320)	0.385	-	-	1.236 (0.716–2.135)	0.446	2.532 (1.340–1.784)	0.004 *
Age	0.979 (0.959–1.000)	0.052	-	-	0.980 (0.956–1.004)	0.105	0.979 (0.955–1.005)	0.107
Albumin	0.646 (0.378–1.102)	0.109	0.567 (0.328–0.982)	0.043 *	1.210 (0.657–2.228)	0.540	1.462 (0.736–2.905)	0.278
eGFR	0.927 (0.908–0.946)	<0.001 *	0.919 (0.899–0.940)	<0.001 *	-	-	0.921 (0.897–0.946)	<0.001 *
Body mass index	1.005 (0.951–1.062)	0.865	0.984 (0.929–1.043)	0.595	1.016 (0.964–1.071)	0.544	1.031 (0.976–1.089)	0.272
Diabetes	1.638 (0.984–2.729)	0.058	-	-	1.521 (0.866–2.673)	0.145	1.901 (1.041–3.473)	0.037 *
Hypertension	1.506 (0.686–3.307)	0.307	1.463 (0.655–3.269)	0.353	1.264 (0.567–2.833)	0.570	0.970 (0.432–2.178)	0.942
Coronary artery disease	0.662 (0.352–1.248)	0.202	0.696 (0.368–1.316)	0.265	0.598 (0.306–1.168)	0.132	0.606 (0.305–1.203)	0.152
Congestive heart failure	0.419 (0.131–1.337)	0.142	0.437 (0.136–1.403)	0.164	0.360 (0.108–1.198)	0.096	0.176 (0.050–0.618)	0.176
Cerebrovascular disease	0.704 (0.172–2.881)	0.625	0.727 (0.176–3.003)	0.107	1.200 (0.279–5.160)	0.806	1.786 (0.406–7.862)	0.443
Peripheral artery disease	1.719 (0.236–12.534)	0.593	1.862 (0.252–13.754)	0.542	1.803 (0.235–13.818)	0.570	1.576 (0.202–12.320)	0.664

Abbreviations: HR, hazard ratio; CI, confidence interval; Model 1: adjusted for age, gender, and diabetes. Model 2: adjusted for all variables, except eGFR. Model 3: adjusted for all other variables. *: Statistically significant, ^#^: Cut-off value obtained from receiver operating characteristic curve by Youden index.

## Data Availability

Not applicable.

## Supplementary Materials

The following supporting information can be downloaded at: https://www.mdpi.com/article/10.3390/nu14153063/s1, Table S1: The parameter, regression equation, correlation coefficient, linear range, and recovery of the five trace elements
