# Supplementary material for: Micronutrients and Renal Outcomes: A Prospective Cohort Study
Source: Nutrients. 2022 Jul 26;14(15):3063. doi: 10.3390/nu14153063 (PMC9370256; doi:10.3390/nu14153063)
Supplement: Supplementary file 1 [file nutrients-14-03063-s001.zip › nutrients-1810888-supplementary.pdf]

Table S1. The parameter, regression equation, correlation coefficient, linear range, and recovery of the five trace elements

Element	Mass (amu)	Mode	CH_4 (mL/min)	RPq	Regression equation	Linear range (ng/mL)	Correlation coefficient	Recovery (%)
Cu	65	Standard	-	-	y = 0.022x + 0.000	0.025-50	0.9999	96.3± 17.1
Zn	67	Standard	-	-	y = 0.001x + 0.000	0.250-50	0.9999	90.0±34.8
Cr	52	DRC	0.8	0.5	y = 0.077x – 0.001	0.025-10	0.9999	88.7±3.5
Mn	55	DRC	0.8	0.5	y = 0.104x -0.003	0.025-5	0.9999	93.3±14.2
Se	78	DRC	0.3	0.45	y = 0.001x – 0.000	0.005-10	0.9998	97.3±17.8

Abbreviations: DRC, dynamic reaction cell
